# Investigation of Pharmacological Activity of *Caralluma penicillata*: Anti-Inflammatory Properties and Gastritis Protection against Indomethacin in Adult Guinea Pigs

**DOI:** 10.1155/2014/738493

**Published:** 2014-12-04

**Authors:** Nabil Albaser, Najeeb Ghanem, Mohanad Shehab, Adnan Al-Adhal, Mohammed Amood AL-Kamarany

**Affiliations:** ^1^Department of Pharmacology and Therapeutic, Faculty of Medicine and Health Sciences, Sana'a University, Sana'a, Yemen; ^2^Tihama Foundation for Drug Studies and Research, Hodeidah, Yemen; ^3^Department of Pharmacy Practice, Faculty of Clinical Pharmacy, Hodeidah University, P.O. Box 3114, Hodeidah, Yemen

## Abstract

*Caralluma* is a plant that possessing a great therapeutic potential in folk medicine in Yemen, namely, *Caralluma penicillata * (*C. penicillata*) as antiulcer. The study aims to evaluate the anti-inflammatory properties and gastritis protection activity of *C. penicillata* against indomethacin in adult guinea pigs. The study was divided into four parts: firstly, the optimum dose of extract as anti-inflammatory effect was determined. Secondly, the acute anti-inflammatory effect of extract were estimated. Thirdly, the repeated doses of extract against chronic inflammation was estimated. The anti-inflammatory activity of extract was compared with indomethacin as a prototype of drug against inflammation. Fourthly, the gastritis protection properties of extract with/without indomethacin were performed. The results showed that a 400 mg/kg of 10% ethanol extract produced the maximum of anti-inflammatory effect. Also, the single dose of extract was equipotent for indomethacin (10 mg/kg), but shorter in duration with regard to acute anti-inflammatory effect. In addition, the repeated doses of extract against chronic inflammation were less potent than indomethacin with regard to ulcerogenic effect. On the other hand, extract-indomethacin combination reduced the gastritis effect of indomethacin based on ulcer index and histological study.

## 1. Introduction

The genus* Caralluma* (*C.*) belongs to the family Asclepiadaceae (also known as the milk weed family) which comprises some 200 genera and 2500 species [1]. It is a plant species possessing a great therapeutic potential in folk medicine. The medicinal properties of* C. *species include antihyperglycemic activity of* C. attenuata *[2], while* C. tuberculata* also offered protection against mucosal damage of stomach [3]. Plants belonging to this genus are rich in esteried polyhydroxypregnane glycosides, some of which showed antitumor activity and others were postulated as precursors of cardenolides [4, 5].* C. arabic*a and* C. umbellate *have shown antinociceptive and anti-inflammatory properties [6, 7]. The use of* C.* species in traditional medicinal was recorded in many studies and* C. penicillata* has been used in Yemeni traditional medicine for the treatment of peptic ulcer and as being antihyperglycemic. Also, some populations used it for the treatment of snake and scorpion bites [8]. The plant, particularly,* C. negevensis, *is also used by Bedouins to treat chronic lung diseases, such as tuberculosis and cancer [1]. However, the stem juice of* C. umbellate *warmed and mixed with turmeric powder is given in stomach disorders and abdominal pain [9]. Also* C. arabica *is commonly used in folk medicine as decoction as being antipyretic and antirheumatic [6].

Several active substances were isolated from various members of* C. *species such as pregnane glycosides and flavonoids. Pregnane glycosides are named* Carambelloside* I and II, which were isolated, particularly, from* C. umbellate *[10] and another twenty new pregnane glycosides were isolated from the whole plant of* Caralluma negevensis *[11]. Megastigmane glycosides and flavonol were isolated from* C. retrospiciens* [1]. However, one of these flavone glycosides is named luteolin-4-O-neohesperidoside which was isolated from another species [2]. Pregnane ester glycosides named caretroside A and biocide were isolated from* C. retrospiciens* [12]. Oxypregnane glycosides are named penicillosides A-G, which were isolated from* C. penicillata *[13, 14]. The most common* C. *species in Yemen is* C. penicillata *that widely is used in folk medicine having an antiulcer effect.* C. penicillata* has been used in Yemeni traditional medicine for the treatment of peptic ulcer and as anti-inflammatory. No scientifically based study was performed on effects of this plant. Therefore this study aimed to evaluate the pharmacological activity of* C. penicillata*, namely, anti-inflammatory properties and gastritis protection against indomethacin in male adult guinea pigs.

## 2. Material and Methods

### 2.1. Standards, Reagents, and Plant Materials

Indomethacin was obtained from MSD (UK) in powder form (secondary standard). Diazepam of Hoffman La Rhoche (Switzerland) in injectable form was purchased. Polysorbate 80 USP was purchased (Sigma Chemical Co. Germany). Ethanol of Merck, Darmstadt (Germany), was purchased. Fresh egg albumin was prepared before use.

### 2.2. Extraction Assay

The aerial parts of the* C. penicillata*, weighing 5 kg, were chopped and crushed in mixer and extracted with 10% ethanol at room temperature. The extract was dried by using freeze dryer.

### 2.3. Animal Handling

Adult male guineas pigs with uniform locally bread strain that weighing from 350 g to 405 g were used throughout this study. They were chosen since they proved most to be convenient for the purpose of the study besides their availability, ease of handling, and cheapness. The choice of male guinea pig was undertaken to avoid interaction with female sex hormones and in order to eliminate the influence of estrous cycle and pregnancy in female guinea pig on tested parameters. Before starting work, the animals were left for one week to acclimatize. They were kept at constant temperature and allowed for food and water ad libitum. They were fed on grass and carrots.

### 2.4. Study Design

This pilot study was conducted in four parts to determine the anti-inflammatory activity of* C. penicillata* selected from Yemen where it remains the most frequently used traditional therapeutic to treat peptic ulcer. The first part was devoted to determining the anti-inflammatory dose of extract. The second part, the acute anti-inflammatory effect of extract, was performed and compared with indomethacin as a prototype of drug against inflammation. The third part, the repeated doses of extract, was estimated against chronic inflammatory and compared with indomethacin; the fourth part, the gastritis protection activity of extract against indomethacin, was estimated.

### 2.5. Extract and Indomethacin Preparations

The dried extract (85 g) was resuspended with distilled water for pharmacological studies. The doses of* C. penicillata* extract were determined according to the pilot study. In this work, indomethacin was dissolved in 5% Polysorbate 80 (10 mg/mL). It was freshly prepared before being used every day [15].

### 2.6. Induction of Inflammation

#### 2.6.1. Induction of Acute Inflammation

Hind paw oedema inflammation in adult male guinea pigs was conducted using a modification of the method described [16]. As described by Akah and Nwambie 1994 [17], 0.5 mL/kg of fresh egg albumin was injected subcutaneously into the right hind paw of each guinea pig under the subplantar aponeurosis. The paw volume was measured at half an hour after the injection using a plethysmograph by dipping the foot in the mercury bath of the plethysmograph apparatus up to the anatomical hairline on lateral malleolus. The inhibitory activity was calculated according to the following formula [18]:

(1) Anti-inflammation  activity=(%  Inhibition)=1−DC×100,

where *D* represents the percentage difference in paw volume after injection of the extract and *C* represents the percentage difference of volume in the control group. On the other hand, for the animals to be handled easily, all received diazepam (2.5 mg/kg) intraperitoneally 30 minutes before inducing edema as a moderate sedative [19].

#### 2.6.2. Induction of Chronic Inflammation

Chronic inflammation was induced by cotton pellet implantation [20], this protocol was described by Sheth et al., 1972 [21] with slight modification to be used for adult male guinea pigs. The animals were fasted 12 hours prior to implantation. Sterile pellets of pure cotton of average weight (50 mg ± 1) were used in this experiment. They were sterilized by heat before implantation. The groin region of all animals was shaved and sterilized by alcohol. A median 2.5 cm subcutaneous incision was performed in groin of each animal under light ether anesthesia. Two of previous prepared cotton pellets were subcutaneously implanted on each side of midline through the incision. It was then sutured by silk threads. The sutured incisions were frequently sterilized by alcohol. After 8 days of cotton pellet implantation, all animals were sacrificed and the cotton pellets were extracted, dried at 70°C, and weighted. The increase in weight of cotton pellet was considered an indicator of chronic inflammation.

### 2.7. Anti-Inflammatory Assay of* C. penicillata*

#### 2.7.1. Determination of Optimum Dose of* C. penicillata* Extract

It was devoted to evaluating the anti-inflammatory effect of* C. penicillata* and to find out the effective dose of 10% ethanol extract for further use in experimental studies. As an initial pilot experiment, increasing doses of* C. penicillata *(200, 400, and 600 mg/kg) were administered intragastrically and studied in the presence of fresh egg albumin-induced hind paw oedema in male adult guinea pigs. Twenty-four guinea pigs were used in this experiment, they were divided into 4 groups, and each group consisted of six animals. The first group served as control and they received oral single 5% Polysorbate 80 in comparable amount to tested drug and plant extract one hour before induction of egg albumin for acute inflammation. Respectively, the second, third, and fourth groups served to study the effect of 200 mg/kg, 400 mg/kg, and 600 mg/kg of 10% ethanol extract of* C. penicillata *on fresh egg albumin induced paw oedema and the plant extract was administered by intragastric tube. The volume of paw oedema in each group was determined with plethysmograph apparatus and the subsequent readings were taken at an interval of 30 min for a total of 180 min. The minimal dose that produced maximal activity effect was found and used throughout the second part of experiment.

#### 2.7.2. Effect Determination of* C. penicillata* Extract on Acute Inflammation Model

It was devoted to investigating the effect of acute intragastric administration of* C. penicillata *on fresh egg albumin induced hind paw oedema in guinea pigs by injecting 0.5 mL/kg of fresh egg albumin into the subplantar surface of the right hind paw. Eighteen adult male guinea pigs were used in this experiment. They were divided into 3 groups each consisting of 6 animals. The first group served as control and they received single 5% Polysorbate 80 intragastrically in comparable volumes to drugs used. The second group served to study the effect of* C. penicillata *extract and they were given oral single extract of* C. penicillata* in a dose of 400 mg/kg. The third group served to study the effect of indomethacin and they received oral single indomethacin in a dose of 10 mg/kg. Guinea pig hind paw oedema was induced by subplantar injection of fresh egg albumin (0.5 mL/kg), using a syringe of 1 mL. Acute inflammation of the hind paw was induced in each of the guinea pigs by injecting 0.5 mL/kg of fresh egg albumin into the subplantar surface of the right hind paw 1 hr after drugs administration. Volume of the hind paw was assessed during the period of 3 hr at 30 min interval after the injection of the inflammatory agent. Increase in the volume of right hind paws was taken as an indication of paw oedema. Oedema was assessed in terms of the difference in original volume of the right hind paw before the injection of inflammatory agent and its volume at time (30, 60, 90, 120, 150, and 180 min) following fresh egg albumin administration. The increase in the right hind paw (induced by the injection of fresh egg albumin) was compared with the original volume of the right hind paw before oedema induction.

#### 2.7.3. Effect Determination of* C. penicillata* Extract on Chronic Inflammation Model

It was devoted to investigating the effect of* C. penicillata *on cotton pellet induced granuloma [22]. A model of chronic inflammation was carried out by implantation of sterile cotton pellets (50 ± 1 mg) in guinea pig paw. Thirty guinea pigs were used in this experiment. They were divided into 5 groups each consisting of 6 animals. The first group served as control and the animals received 5% Polysorbate 80 single intragastrically in comparable volumes to drugs used for 7 consecutive days from the day of cotton pellet implantation. The second group served to study the effect of* C. penicillata*; the animals were given oral dose of* C. penicillata *(400 mg/kg) once daily at 8 PM for 7 consecutive days from the day of cotton pellet implantation. The third group served to study the effect of indomethacin as a test reference and the animals were given oral dose of indomethacin (10 mg/kg) once daily as aqueous suspensions using 5% Polysorbate 80 by [23] at 8 PM for 7 consecutive days from the day of cotton pellet implantation. The fourth group served to study the effect of combination dose of* C. penicillata *and indomethacin and the animals were given oral dose of indomethacin (5 mg/kg) and* C. penicillata *(200 mg/kg) once daily at 8 PM for 7 consecutive days from the day of cotton pellet implantation. The fifth group served to study the effect of combination dose of* C. penicillata *and indomethacin and the animals were given oral dose of indomethacin (10 mg/kg) and* C. penicillata *(400 mg/kg) once daily at 8 PM for 7 consecutive days from the day of cotton pellet implantation. The combination doses of* C. penicillata *and dose of indomethacin were administered to animals groups separately with 30 minutes between them.

The same volume of distilled water was applied to the control group. After 30 min, a model of chronic inflammation was induced in all animals; the animals were anesthetized with light ether. Under sterile conditions, cotton pellets, weighing (50 mg ± 1) each, were implanted an interscapular distance under the skin, according to the method of Swingle and Shideman, 1972 [24]. After 7 days of drugs administration all animals were sacrificed by decapitation.

### 2.8. Antiulcerogenic Assay of* C. penicillata*

They were dissected for stomach and cotton pellets. Cotton pellets were removed, dried, and weighed. The stomachs were cut open along the greater curvature rinsed with normal saline for the scoring of ulcer index. In addition, they were kept in 10% formalin before histological examinations were performed. The stomach tissues were subjected to normal routine histological procedures, stained with hematoxylin-eosin and examined using the light microscopy for any morphological changes [25–27].

### 2.9. Data Analysis

Data obtained were analysed by using descriptive analysis, namely, mean and standard error (SE). Also, Student's *t*-test was used to explore the anti-inflammatory and antiulcerogenic properties of* C. penicillata* and for comparison with indomethacin.

## 3. Results

### 3.1. Anti-Inflammatory Dose of* C. penicillata* Extract

**Figure 1:**
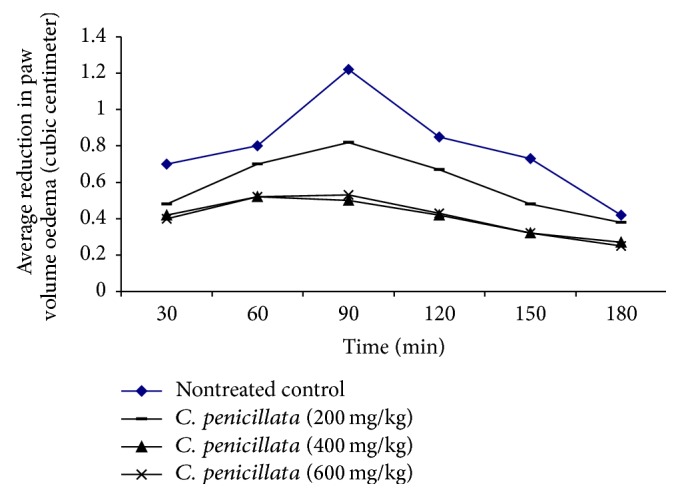
The maximum anti-inflammatory effect of different intragastric doses of 10% ethanol extract of* C. penicillata* admitted singly on average (M ± SE) paw volume oedema (cubic centimeter) of fresh egg albumin (0.5 mL/kg) induced paw oedema in adult male guinea pigs (*n* = 6) after 30, 60, 90, 120, 150, and 180 minutes and compared with nontreated control group.

The Anti-inflammatory effect of single intragastric administration of different doses of* C. penicillata *on fresh egg albumen induced paw oedema in adult male guinea pigs to find out the effective dose for further use in experimental studies. The normal volume of hind paw of adult male guinea pig was 3.15 ± 0.10 cc. Induction of acute inflammation by injecting fresh egg albumin (0.5 mL/kg) into subplantar aponeurosis of the right hind paw increased its volume with the time (Table 1). A dose of 200 mg/kg of 10% ethanol extract of* C. penicillata* significantly reduced (*P* < 0.05) the average volume of egg albumen induced oedema with the time compared with the original paw volume and nontreated control group except at 180 min for nontreated control group in which no statistically significant difference was observed (*P* > 0.05). This may imply partial improvement of acute egg albumen induced oedema after the administration of this extract dose. The dose of 400 mg/kg of extract was more effective than the previous dose (200 mg/kg). It significantly reduced the volume of paw oedema more than 200 mg/kg with the time compared with the original paw volume (*P* < 0.05) and nontreated control group. The administration of 600 mg/kg of extract significantly reduced (*P* < 0.05) the volume of paw oedema with the time compared with the original volume and nontreated control group. Also final dose had the same effect of 400 mg/kg. Economically, this means that the most effective dose of extract was 400 mg/kg (Figure 1).

### 3.2. Effect of* C. penicillata* on Acute Inflammation Model

The results of anti-inflammatory activity of* C. penicillata* were summarized in Table 2. The intragastric administration of single dose of* C. penicillata *(400 mg/kg) significantly reduced (*P* < 0.05) the average volume of paw oedema by 59%, 51%, 56%, and 36% after 90, 120, 150, and 180 minutes, respectively, compared with the original volume of paw. Also, the intragastric administration of indomethacin (10 mg/kg) significantly reduced (*P* < 0.05) the average volume of the paw oedema by 61%, 65%, 77%, and 71% after 90, 120, 150, and 180 minutes, respectively, compared with the original volume of paw. On the other hand, statistically significant difference (*P* < 0.05) was observed between nontreated control group and tested and indomethacin group. In addition,* C. penicillata *had 40%, 88%, and 125% less than anti-inflammatory effect of indomethacin after 120, 150, and 180 minutes, respectively; on the other hand, significant difference (*P* < 0.05) was observed between both groups at the same time (Table 2).

### 3.3. Effect of* C. penicillata* on Chronic Inflammation Model

Table 3 showed the anti-inflammatory effect of repeated intragastric administration of* C. penicillata* (400 mg/kg) and indomethacin (10 mg/kg) on cotton pellet induced granuloma as a model of chronic inflammation; significant difference results (*P* < 0.05) between both groups were summarized in this table. On the other hand, implantation of 50 mg ± 1 cotton pellet in male adult guinea pig caused in the formation of the granulomatous chronic inflammation weighed 83.9 ± 3.2 mg. The indomethacin 10 mg/kg and* C. penicillata *400 mg/kg produced significant anti-inflammatory effect (*P* < 0.05) that was evident in the reduction of the weight of cotton pellet induced granuloma by 74% and 64%, respectively, compared with nontreated control group. Also, the combination of the above mentioned agents in half doses reduced the weight of cotton pellet induced granuloma by 57% compared with nontreated control and significant difference was recorded (*P* < 0.05). In addition, the combination of full dose of both agents produced the same quantitative effect; on the other hand, it reduced the cotton pellet induced granuloma by 49% compared with nontreated control group. These results may indicate that the extract has less anti-inflammatory effect than indomethacin. In addition, there was minor change with regard to the anti-inflammatory effect between half and full doses of both agents.

### 3.4. Antiulcerogenic Properties of* C. penicillata* and Gastritis Protection against Indomethacin

**Figure 2:**
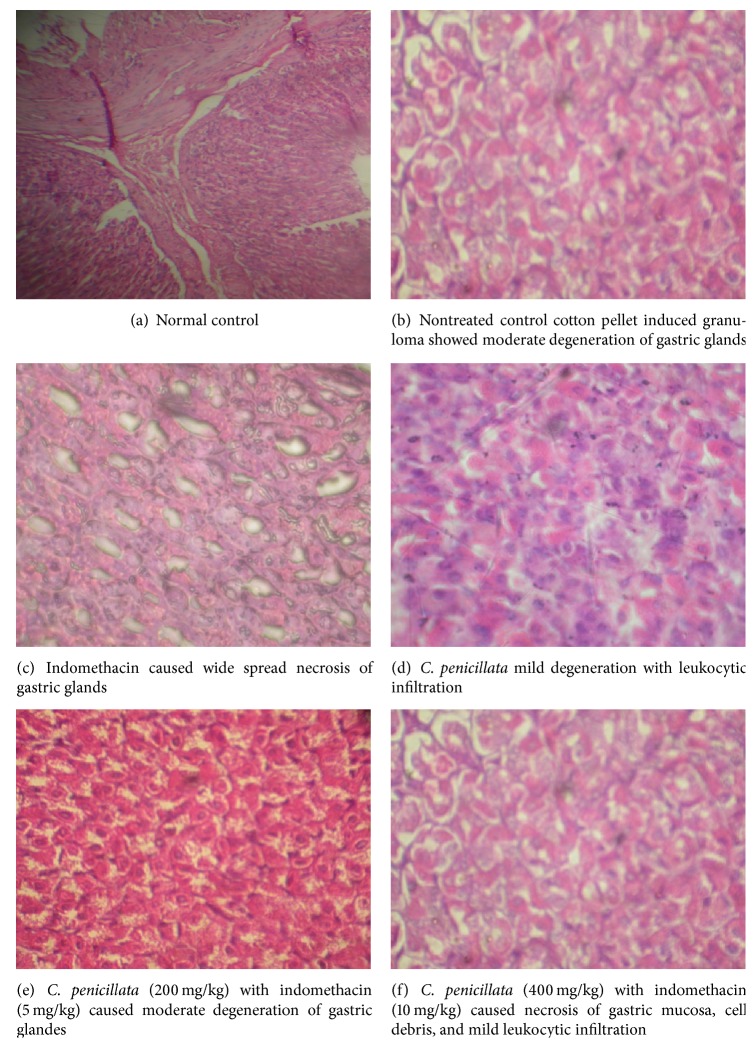
Histopathological study of normal control, nontreated control, indomethacin,* C. penicillata*, and half and full doses combination.

*C. penicillata * 400 mg/kg or indomethacin 10 mg/kg singly or in combination in half or full doses showed significant ulcerogenic potential evidenced in raising of ulcer score by +50%, +250%, +150%, and +167% compared with normal control group, respectively (Table 4). The combination of* C. penicillata *with indomethacin reduced the ulcerogenic effect of indomethacin by 28% in case of half dose combination and by 24% in case of full dose combination compared with indomethacin. On the other hand, Figure 2 showed histopathological study of six groups that indicated the following: (a) normal control, (b) nontreated control “cotton pellet induced granuloma” that showed moderate degeneration of gastric glands, (c) treated group with indomethacin (10 mg/kg) that caused the wide spread necrosis of gastric glands, (d) treated group with* C. penicillata* that caused the mild degeneration with leukocytic infiltration, (e) treated group with* C. penicillata* (200 mg/kg) and indomethacin (5 mg/kg) as half combination form that caused moderate degeneration of gastric glands, and (f) treated group with* C. penicillata* (400 mg/kg) and indomethacin (10 mg/kg) as full combination form that caused necrosis of gastric mucosa, cell debris, and mild leukocytic infiltration.

## 4. Discussion

This study included testing of 10% ethanol extract of* C. penicillata *on two models of experimentally induced inflammation, namely, the fresh egg albumen induced paw oedema and production of granuloma by subcutaneous implantation of cotton pellet. The pathogenesis of both methods depends on the reaction of the body to foreign materials. In the former method 0.5 mL/kg of fresh egg albumen was injected subplantarly. It acts as irritant foreign body which induced acute phase inflammatory reaction of short duration. It is characterized by the exudation of fluids and plasma proteins and emigration of leukocytes mainly neutrophil from the blood vessels and perivascular tissues to the site of foreign body implantation. The latter is mediated by interaction between complementary adhesion molecules present in leukocytes and endothelial surface. The expression of adhesion molecules is modulated by inflammatory agents. The volume of exudates is directly proportional to severity of acute inflammation [28]. On the other hand, the implantation of foreign body for relatively longer time as in case of cotton pellet implantation model of chronic inflammation promotes the formation of granuloma. It is a tumor like structure composed of macrophages, fibroblasts, and capillaries in varying amounts depending on the type of implanted substances. The weight of the granuloma is proportional to severity of inflammatory response [28].

The appropriate anti-inflammatory dose of extract was 400 mg/kg of 10% ethanol extract of the tested plant produced the optimum and maximal effect. The present work revealed that the extract produced equipotent anti-inflammatory effect to an intragastric dose of indomethacin amounted to 10 mg/kg for the first 90 minutes and then indomethacin was superior to the tested extract in the last 90 minutes of experimental period. This may imply that the tested extract had shorter duration of action than indomethacin that acts by inhibition of inflammatory pathway of inflammatory prostaglandin synthesis by inhibition of cyclooxygenase-2. Prostaglandins E1, E2, and D series are potent inflammatory mediators. They mediate acute inflammatory response through increasing capillary permeability and endothelial dysfunction [29].

The anti-inflammatory effect of extract is in conformation with the previous reports for other species of this plant. Ramesh et al. showed that either orally administrated extract of* C. arabica* in a dose of 200–400 mg/kg or topical 5% gel reduced carrageenan induced paw oedema [7]. Moreover, acute oral dose of the previously mentioned plant extract produced significant antinociceptive effect on various models of pain, namely, tail flick test, abdominal constriction test, and hot plate test. It is hard to define the mechanism of such effect and it needs a detailed phytochemical study to identify the active constituents of extract. Nevertheless, it may be assumed that the high flavonoids, sterols, and triterpenes of extract are the active ingredients responsible for the anti-inflammatory effect. In 1999, Qiu et al. isolated a common flavonoid glycoside, luteolin-4-O-neohesperidoside in most* C*. genera [30]. Ramesh et al. in 1998 isolated the above mentioned flavonoid from* C. attenuate*. A dose of 3-4 mg/kg was used as potent anti-inflammatory comparing to a 50 mg/kg of Ibuprofen. Higher doses (100 mg/kg) exerted antinociceptive effects. It may exert the anti-inflammatory effect by acting as free radical scavenger [31]. Ansari et al., 2005, showed a potent positive correlation between the antioxidant effect and the biological effects of* Caralluma *species and oxidative free radicals are highly reactive substances produced by granulocytes in response to engulfing of foreign materials [32].

Compared with 10 mg/kg intragastric indomethacin, the tested extract produced less potent anti-inflammatory effect. Indomethacin-*C. penicillata *combination antagonized each of them regarding the anti-inflammatory effect. This may be explained by pharmacokinetic antagonism as a result of the combined agents with regard to their gastrointestinal absorption. Indomethacin is weakly acidic drug related to indole acetic acid group [33]. The weakly acidic drugs like indomethacin are less ionized and highly absorbable in acidic media. Coadministration of* C. penicillata *and indomethacin may inhibit gastrointestinal absorption of indomethacin by reducing gastrointestinal acidity through its high mineral basic substances as well as simple volume dilution effect [34]. Concerning the effect of the tested agents on gastric mucosa, either indomethacin or* C. penicillata *significantly raised ulcer index compared with normal control and nontreated groups. This may indicate that the tested agents have a marked ulcerogenic potential. This may be attributed to the effect of indomethacin in decreasing synthesis of protective prostaglandins in gastric mucosa. The latter exerts cytoprotective effect by increasing mucous secretion and vasodilatation of mucous blood vessels. It is worth mentioning that indomethacin inhibits cyclooxygenase-I responsible for synthesis of physiological prostaglandins compared to cyclooxygenase-II that is responsible for synthesis of inflammatory prostaglandin [35]. On the other hand, the ulcerogenic effect of* C. penicillata *was hard to be explained from the available data and combination of* C. penicillata *in full dose significantly reduced the ulcerogenic effect of indomethacin. This may be attributed to formation of inactive complex of the two agents in gastric mucosa. The coadministration of both agents in half the therapeutic dose significantly reduced the ulcerogenic potential and anti-inflammatory effect compared with indomethacin alone but it is more toxic and as effective compared with* C. penicillata *alone. The above mentioned combination is as effective as and less toxic than combining the full therapeutic dose of both agents. These data may imply that the modest anti-inflammatory effect and the least ulcerogenic effect can be obtained by combining half the therapeutic dose of both indomethacin and* C. penicillata*. This is in conformation with the work of Al-Harbi et al. who showed that* C. tuberculata *at doses of 250, 500, and 1000 mg/kg provided dose-dependent protection against the damage caused by 80% ethanol, 0.2 M NaOH, hypertonic saline, and indomethacin and it can be assumed that the* C. *species has both ulcerogenic and cytoprotective effects; the former effects predominated when the drug was taken alone. This may be due to sterols content. On the contrary, the cytoprotective effect predominated when the plant is combined with ulcerogenic agents like indomethacin. This may be due to increase of production of mucous and prostaglandin, free radical scavenging, and sulfhydryl compound spearing effects [3]. Finally, the ulcerogenic potential of tested agents observed in this work was well correlated with microscopic study of gastric mucosa. The latter revealed that cotton pellet implantation caused mild degeneration changes in gastric glands. Probably due to immune mediated mechanism triggered by foreign implantation, doses of* C. penicillata *produced mild degeneration with leukocytic infiltration mucosa. Indomethacin alone produced wide spread necrosis of gastric mucosa with accumulation of cell debris in gastric glands. These changes persisted but in milder form when indomethacin was admitted in combination with* C. penicillata*. This result indicated partial activity of extract to protect the mucosa from side effect of indomethacin.

In conclusion, the extract of* C. penicillata* but hadanti-inflammatory effect against acute and chronic models of inflammation. A single dose of 400 mg/kg of the extract was equipotent for efficient indomethacin but shorter action than it. The repeated of dose extract was less effective as anti-inflammatory and ulcerogenic potential than indomethacin. In brief, this study has shown that* C. penicillata *possesses anti-inflammatory and antiulcerogenic properties, thus confirming the claim by traditional medical practitioners. Further studies are necessary in order to determine the active ingredients in the plant and to evaluate the specific action mechanism of this plant.

## Figures and Tables

**Table 1:** The anti-inflammatory effect of single intragastric administration of different doses of *C*. *penicillata* on fresh egg albumen (0.5 mL/kg) induced paw oedema in adult male guinea pigs (*n* = 6) to find out the effective dose for further use in experimental studies.

Groups	Original paw volume	Average volume of paw (M ± SE) paw volume (cubic centimeter)					
		30 min	60 min	90 min	120 min	150 min	180 min
Nontreated control Difference of paw volume (cc)	3.15 ± 0.10	3.82 ± 0.13^*^	3.95 ± 0.13^*^	4.35 ± 0.14^*^	4.0 ± 0.16^*^	3.9 ± 0.15^*^	3.57 ± 0.14^*^
		0.7 ± 0.04	0.80 ± 0.04	1.2 ± 0.03	0.85 ± 0.05	0.75 ± 0.04	0.42 ± 0.03

*C*. *penicillata * (200 mg/kg) Difference of paw volume (cc)	3.08 ± 0.07	3.53 ± 0.09^*^	3.78 ± 0.09^*^	3.9 ± 0.10^*^	3.75 ± 0.10^*^	3.57 ± 0.09^*^	3.47 ± 0.09^*^
		0.45 ± 0.03^+^	0.70 ± 0.02^+^	0.82 ± 0.03^+^	0.67 ± 0.04^+^	0.49 ± 0.05^+^	0.39 ± 0.03^+^

*C*. *penicillata* (400 mg/kg) Difference of paw volume (cc)	3.15 ± 0.10	3.57 ± 0.11^*^	3.67 ± 0.09^*^	3.65 ± 0.10^*^	3.57 ± 0.11^*^	3.47 ± 0.11^*^	3.42 ± 0.09^*^
		0.42 ± 0.03^+^	0.52 ± 0.04^+^	0.50 ± 0.05^+^	0.42 ± 0.03^+^	0.32 ± 0.03^+^	0.27 ± 0.03^+^

*C*. *penicillata* (600 mg/kg) Difference of paw volume (cc)	3.0 ± 0.08	3.40 ± 0.06^*^	3.52 ± 0.11^*^	3.53 ± 0.11^*^	3.33 ± 0.16^*^	3.32 ± 0.11^*^	3.25 ± 0.05^*^
		0.40 ± 0.02^+^	0.52 ± 0.03^+^	0.53 ± 0.03^+^	0.33 ± 0.02^+^	0.32 ± 0.02^+^	0.25 ± 0.02^+^

^*^Significantly compared with original paw volume at *P* < 0.05.

^+^Significantly compared with nontreated control group at *P* < 0.05.

cc: cubic centimeter.

**Table 2:** Effect of single dose intragastric administration of 10% ethanol extract of *C. penicillata* (400 mg/kg) versus indomethacin (10 mg/kg) on average (M ± SE) volume (cubic centimeter) of fresh egg albumin (0.5 mL/kg) induced paw oedema as a model of chronic inflammation (*n* = 6).

Groups	Original paw volume	Average volume of paw (M ± SE) paw volume (cubic centimeter)					
		30 min	60 min	90 min	120 min	150 min	180 min
Nontreated control Difference of paw volume (cc)	3.15 ± 0.10	3.82 ± 0.13^*^	3.95 ± 0.13^*^	4.35 ± 0.14^*^	4.0 ± 0.16^*^	3.9 ± 0.15^*^	3.57 ± 0.14^*^
		0.7 ± 0.04	0.80 ± 0.04	1.2 ± 0.03	0.85 ± 0.05	0.75 ± 0.04	0.42 ± 0.03

Indomethacin (10 mg/kg) Difference of paw volume (cc)	3.18 ± 0.11	3.68 ± 0.12^*^	3.78 ± 0.11^*^	3.65 ± 0.08^*^	3.48 ± 0.04^*^	3.35 ± 0.10	3.30 ± 0.1^+^
		0.50 ± 0.05^+^	0.60 ± 0.03^+^	0.47 ± 0.03^+^	0.30 ± 0.03^+^	0.17 ± 0.02^+^	0.12 ± 0.01^+^

*C. penicillata* (400 mg/kg) Difference of paw volume (cc)	3.15 ± 0.10	3.57 ± 0.11^*^	3.67 ± 0.09^*^	3.65 ± 0.10^*^	3.57 ± 0.11^*^	3.47 ± 0.11^*^	3.42 ± 0.09^*^
		0.42 ± 0.03^+^	0.52 ± 0.04^+^	0.50 ± 0.05	0.42 ± 0.03^+*ϵ*^	0.32 ± 0.03^+*ϵ*^	0.27 ± 0.03^+*ϵ*^

^*^Significantly compared with original paw volume at *P* < 0.05.

^+^Significantly compared with nontreated control group at *P* < 0.05.

^*ϵ*^Significantly compared with indomethacin group at *P* < 0.05.

**Table 3:** Anti-inflammatory effect of repeated intragastric administration of *C*. *penicillata* extract (400 mg/kg) and indomethacin (10 mg/kg) either singly or in combination on cotton pellet induced granuloma as a model of chronic inflammation (*n* = 6).

Groups	Nontreated control	Indomethacin (10 mg/kg)	*C. penicillata* (400 mg/kg)	Half dose combination	Full dose combination
Dry granuloma (mg)	83.9 ± 3.2	22.9 ± 2.8^+^	30.6 ± 1.5^+*ϵ*^	36 ± 3.0^+*ϵ*^	43 ± 1.8^+*ϵ*^

Half dose combination: *C*. *penicillata* (200 mg/kg) with indomethacin (5 mg/kg); full dose combination: *C*. *penicillata* (400 mg/kg) with indomethacin (10 mg/kg).

^+^Significantly compared with nontreated control group at *P* < 0.05.

^*ϵ*^Significantly compared with indomethacin group at *P* < 0.0.

**Table 4:** Effect of repeated intragastric administration of *C*. *penicillata* extract (400 mg/kg) and indomethacin (10 mg/kg) either singly or in combination in cotton pellet induced granuloma in adult male guinea pigs (*n* = 6) to find out the the ulcer index (M ± SE).

Groups	Normal control	Nontreated control	Indomethacin (10 mg/kg)	*C*. *penicillata* (400 mg/kg)	Half dose combination	Full dose combination
Ulcer index	1.2 ± 0.10	1.5 ± 0.05^*^	4.2 ± 0.31^∗+^	1.8 ± 0.11^∗+^	3.0 ± 0.27^∗+*ϵ*^	3.2 ± 0.28^∗+*ϵ*^

^*^Significant compared with normal control values at *P* < 0.05.

^+^Significant compared with nontreated control group at *P* < 0.05.

^*ϵ*^Significant compared with indomethacin at *P* < 0.05.
